# Nursing students’ perceived consequences of self-medication: a qualitative study

**DOI:** 10.1186/s12912-020-00460-8

**Published:** 2020-07-25

**Authors:** Alireza Khatony, Ali Soroush, Bahare Andayeshgar, Alireza Abdi

**Affiliations:** 1 Social Development and Health Promotion Research Center, Kermanshah University of Medical Sciences, Kermanshah, Iran; 2 Clinical Research Development Unit, Imam Reza Hospital, Kermanshah University of Medical Sciences, Kermanshah, Iran; 3 Doolat abaad, Nursing Department, School of Nursing and Midwifery, student research committee, Kermanshah University of Medical Sciences, Dolat Abad Street, Kermanshah, Kermanshah province 6718996511 Iran

**Keywords:** Self-medication, Student, Nurse, Qualitative study

## Abstract

**Background:**

Self-medication associates with many problems and complications, and is considered as a global health issue. Regarding a lack of information about perception of nursing students, as a part of healthcare workers, in this issue, the current study was aimed to explore the perceived consequences of self-medication from the perspective of nursing students.

**Methods:**

This qualitative study was conducted using semi-structured individual face-to-face interviews. Purposeful sampling method was used for selection of participants. Twelve nursing students in the age range of 21–36 years were enrolled. Five participants were male and seven were female, seven master’s degree and five were undergraduate of bachelor degree. Data were collected using semi-structured interviews and analyzed as content analyses.

**Results:**

Two categories and seven sub-categories were emerged from the data analysis. The main categories included; positive consequences and negative consequences, and subcategories included; time saving, cost savings, disease treatment, harming the health system, drug resistance, physical complications and death.

**Conclusion:**

The participants believed that self-medication has some positive and negative consequences in viewpoints of nursing students. Regarding, Self-medication that potentially has dangerous consequences, it is suggested the students will be educated and warned about the adverse effects of self-medication, and the nursing teachers should try to rectify students’ misconceptions about self-medication.

## Background

Today, drug abuse and self-medication in general are among the biggest socio-health and economic problems among various societies, including Iran. Research has shown that adverse drug reactions, increased complications, disease coverage, drug interactions, misdiagnosis, and antibiotic resistance are among the adverse self-medication results [1]. Self-medication refers to the use of medication for treatment, which is carried out without prescription or professional counseling [2]. Furthermore, this term relegates to the treatment of self-diagnosis symptoms, the use of previous versions of the same illness and the unusual use of a drug [1, 3]. This practice is a common practice throughout the world [4]. The arbitrary use of medication is common in many societies. The prevalence of self-medication is reported to be 68% in European countries, 77% in USA, 92% in Kuwait, 31% in India and 59% in Nepal [5]. It is also estimated that 83.3% of the Iranian population are taking drugs arbitrarily [6].

Evidence suggests that self-medication is a major problem among university students [7]. Meanwhile, the educated people in society, including university students, especially nursing students are expected to be more aware of the consequences of uncontrolled use of drugs than ordinary people. Arbitrary drug use can have many complications and consequences [8], including misdiagnosis, overdose, long-term use of medication, and drug interactions [9, 10]. In this regard, Zhao et al. in their study referred to the risks of self-medication, which included delaying search for professional counseling, misdiagnosis, rare but severe side effects, drug misuse, incorrect dosage, inaccurate treatment choice, and risk of abuse [11]. The unreasonable use of medication can even lead to death. One study showed that, the simultaneous and arbitrary use of multiple medications causes some disorders in hemostasis, renal function, hemorrhagic stroke and death [12]. In another study, 32.3% of students, who had self-medication, developed side effects [13]. However, there are some controversy opinions about this issue and many medical students believe that self-medication is safe [6] and have a positive attitude towards its effects [14]. They also avoid paying visit to doctor for reasons such as not taking the disease seriously and not having time. Moreover, they try to treat themselves with self-confidence as they think they have high pharmacological knowledge [4].

Regarding the high rate of self-medication among Iranian people, understanding and perception of the individuals about a health issue and the consequences have more priority. Therefore, about self-medication in nursing students (as the big population of healthcare workers), and one of the challenges of health professionals, there is a lack of information. Because the perception and understanding of the results and outcomes of self-medication that is quite subjective, the qualitative research was opted, thus, the current study was conducted for exploring the consequences of self-medication from the perspective of nursing students.

## Methods

### Study design and settings

This study was conducted as a qualitative study from June to September 2017 using inductive content analysis method. This approach has been taken from the naturalistic paradigm, which believes that knowledge is gained from the engagement of the researcher with the participant, and the experience of individual is examined as understandable themes of the phenomenon under study [15]. The settings were, Kermanshah University of Medical Sciences (KUMS) (https://www.kums.ac.ir/en/home) who had about 700 nursing students and graduates about 150 nurse annually.

### Study participants

The study population included all nursing students of KUMS, and the study samples consisted of nursing students with self-medication who were selected purposefully. It is notable; the first and fourth authors are the teachers of the students. Inclusion criteria were students who had self-medication during another cross-sectional research that conducted by the authors of this study [16] on the incidence of self-medication, and consented to participate in the study (by giving the phone number or email in the former questionnaire).

### Data collection procedure

Sampling was done using purposeful method and the nursing students who had experiences of self-medication (based on the previous study in reference [16]) invited to participate in the study by telephone or email. Data were collected using semi-structured interviews and open-ended guide questions that developed by researchers based on literature review and experts opinions, including “Why and how do you self-medicate? What are the benefits and disadvantages of self-medication in your opinion? What factors encourage your self-medication? What factors prevent you from self-medication?” To clarify the concepts, probing questions such as “Please explain more”, “why?” and “how?” were used. This manuscript is a part of a project that the first article and interview guide has published previously [17]. The interviews were conducted by the first and third authors, who are PhD (in nursing education) and MSc (in biostatistics), and they are knowledgeable and expert in qualitative researches and are believed in naturalistic world view. After obtaining permission from the Ethics Committee of Kermanshah University of Medical Sciences, all interviews were conducted at the hospital. The participants were interviewed by the face-to-face method in Persian language, and each interview was carried out in one session. Although, participates had the authority (by informing the researcher) to decline the co-operation in the study, we had no refused to participate or dropped out among them. The minimum interview time was 30 min, and the maximum interview time was 55 min. The data became saturated in the ninth interview which there is no emergence of new themes and concepts, moreover, to ensure this, three other interviews were done. In qualitative studies, data saturation indicates the completion of interviews, which means that previous information is repeated and no new data is generated [18]. We used voice recording to collect data as well as some important notes were written. After each interview, the recorded information was handwritten verbatim and typed.

### Ethical considerations

The present study was approved by the Ethics Committee of Kermanshah University of Medical Sciences with the code: Kums.rec.1396.25. The researcher, after introducing herself to the participants, the study’s objectives and topic were explained to them, and they were assured of the confidentiality of their information and having the authority to reject the invitation or maintain in participation/withdraw from the study. Written informed consent was obtained from all participants.

### Data analysis

Data analysis began simultaneously with the data collection. Data from interviews were analyzed using qualitative content analysis. In this approach, the researcher gains a deep understanding of the concepts related to the subject under study, and codes (317 codes) and sentences are classified into categories and subcategories that make it easier to understand the phenomenon [19]. In the current study, the classification system was inductive. All themes derived from the data. The main idea in this process is to determine the defined criteria that are obtained from the theoretical background and the research question. In this context, the texts of the interviews were examined, the criteria were considered and the categories were gradually emerged step by step [20]. Thus, the texts of the interviews were read several times and a general understanding of their content was obtained, and then the original codes were extracted. The similar codes were placed into a class, and this way, the categories and subcategories were determined. MAXQDA-10 software was used to manage the data.

### Trustworthiness

The Densin and Lincoln criteria were used for data rigor [21].

- A: **Credibility of data:** To ensure data credibility, we tried to interview the participants for a long time, and samples were varied in terms of age and education level. Repeated questions were also used to ensure the participants’ responses. Furthermore, the participants (member check) and colleagues (researchers to do peer check) reviewed the texts, codes and categories of the interviews, as the member check and peer check, respectively.
- B: **Dependability of data:** This indicates that, the findings are accurately aggregated and are not affected by the researchers’ error or interests. In this study, the methods of data collection, interviewing, taking notes, coding, analyzing the data and identifying the content were accurately stated to be judged correctly by the external audit.
- C: **Confirmability:** It refers to the relevance of the concepts and categories, and their proximity to the purpose of the study, and it is measured by the consistency of two independent individuals with regard to the accuracy, relevance and meaning of the data. Since the interviews were conducted by one person, to ensure the confirmability of the study, recorded interviews and their transcripts, together with the coding process, were made available to other colleagues specialized in qualitative research, and the authenticity of the coding was confirmed by them.
- D: **Transferability:** The main goal of qualitative studies is not to generalize results, such as those in quantitative studies. The transferability indicates that the findings are consistent with other contexts. In this study, the phenomenon was studied carefully and the characteristics of the educational situation and the background of the research samples were explained, and the results were presented to the three nursing students who were not among participants, however, they had self-medication and their experiences were compared with the results of present study.

### Findings

The present study aimed to explore the consequences of self-medication from the perspective of nursing students. Twelve nursing students with age 21–36 years-old were enrolled in the study from whom, five were male and seven were females. Also, seven of them were postgraduate and five were undergraduate students (Table 1). It is notable; there was not anyone else present besides the participants and researchers.

**Table 1:** the demographic features of nursing students who participated in the study

Participants	Age (yrs.)	Grade
First	> 30	MSc.
Second	< 30	BSc.
Third	< 30	MSc.
Fourth	< 30	MSc.
Fifth	< 30	MSc.
Sixth	< 30	MSc.
Seventh	< 30	BSc.
Eighth	< 30	BSc.
Ninth	< 30	BSc.
Tenth	< 30	BSc.
Eleventh	> 30	MSc.
Twelve	> 30	MSc.

The qualitative analysis resulted in two main categories and seven sub-categories from 317 codes of data analysis. The main categories included perceived positive and negative consequences, and the sub-categories included time saving, cost savings, disease treatment, harming the health system, drug resistance, physical complications, and death (Table 2).

**Table 2:** categories and subcategories extracted from the data

categories	Sub-categories
**Perceived Positive consequences**	**1.** Time saving **2.** Cost saving **3.** Disease treatment
**Perceived Negative consequences**	**1.** Harming the health system **2.** Drug resistance **3.** Physical complications **4.** Death

#### Perceived positive consequences

From the perspective of most nursing students, in cases of simple and recurrent diseases, there was no need to visit a doctor and they self-medicated using their own and others’ past experiences and knowledge they had about drugs and their side effects. From the perspective of these students, the positive consequences of self-medication included saving time, saving costs and treating the disease. The 192 codes were placed in this category.

##### Time saving

Most participants believed that self-medication saves time, and some believed that a visit to a doctor is a waste of time when the disease is simple and they themselves could treat the disease. Most students believed that, the doctors’ waiting time was long and people had to spend a lot of time visiting a doctor. The 78 codes were placed in this subcategory. One student said, *“I need to spend a lot of time to visit and pay for the doctor, and this is a waste of time, while I can treat myself with my illness with self-medication”* (Eighth interview). Another student said*: “To visit a doctor even a short visit, I must have a full day off, and there is no longer any incentive to go back”* (third interview).

##### Cost saving

Most participants believed that, the cost of treatment is high for the middle-class community and in addition to that, they must pay for the travel costs. They believed that self-medication can be used to save the doctor’s visit and travel costs. Sixty codes were placed in this subcategory. *“I think self-medication is a good thing because it saves the cost of treatment, which is expensive, especially if someone is not insured or is from a middle-class family”* (Second interview). Another student stated: *“Despite the low cost of visits in hospitals, we must still pay for the travel to and from the hospital, so I get the medications and I will not pay for the visit and travel”* (Third interview).

##### Disease treatment

The most important positive consequence of self-medication from nursing students’ viewpoints was the treatment of simple diseases like colds without the need to spend time and money. The 54 codes were placed in this subcategory. One student in this regard stated: *“Sometimes I feel like I have got cold, which is not important and I do not have to visit a doctor for it. Also, if I have already had a disease, such as a common cold, I use my previous experiences with the same drugs, and will be fine”*(Third interview). Another student stated: “*I use painkiller every time I have pain and it is very effective so I do not need to go to a doctor anymore. When I have stomach pain I use a Ranitidine pill, which was previously prescribed by my doctor, and I have seen its effect, so I take it and I’ll be fine”*(Second interview).

#### Perceived negative consequences

Some participants were familiar with the negative consequences of self-medication and experienced some of these consequences. The four sub-categories of the negative consequences of self-medication from the perspective of participants included; harming the health system, drug resistance, physical complications and death. The 125 codes were placed in this category.

##### Harming the health system

The health system is referred to all activities whose main purpose is to promote, restore or maintain health. Some of the participants pointed to the harming of health system as one of the disadvantages of self-medication. The 20 codes were placed in this subcategory. *“When I self-medicate wrongly and do not get a good response, I will have to take the next medication or I might even be admitted to hospital or worse, and all these will impose cost to the health system”*(Twelfth Interview). Another student in this regard stated: *“Among the risks of self-medication is harming the health system ... We work within a system called the health system so we must be careful about everything ... I should not impose a cost on the health system. The health system anywhere in the world has a limited budget and with any self-medication I am reducing some of that budget. So if this self-medication is unscientific and non-principled, it will harm the health system and endangers millions of lives”*(sixth interview).

##### Drug resistance

Most nursing students referred to the adverse effects of self-medication on drug resistance, and referred to the antibiotics as risky drugs. The 55 codes were placed in this subcategory. In this regard, one student stated: *“A person who takes antibiotic arbitrarily, prevents the recovery and cases his/her body to develop resistance against that antibiotic. So when the infection gets exacerbated, the antibiotic does not have any positive effect on him/her body.”* Another student stated: *“I often get cold, I am taking antibiotics myself, and now my body has developed resistance against the antibiotics, so simple antibiotics do not work on me anymore”* (Tenth interview).

##### Physical complications

Some students were familiar with the complications of medications they used and were trying to use medications that had fewer side effects. However, they believed that each drug had its own side effects and stated that, they would stop taking drug in case of side effects. They considered this practice to be similar to the physician’s practice, as even the doctors, when they prescribe medication, they ask the patients to stop taking the medication if they develop side effect and consult a doctor. Some participants were taking medications without knowing their side effects. The 35 codes were placed in this subcategory. One student said: *“I recently suffered from dizziness after taking Azithromycin. However I had experienced these side effects before when doctor prescribed the same medication for me, so I do not think I am having these side effects just because of self-medication, but the side effects are because of the antibiotics”*(Fourth Interview). Another student stated: “*I used the Zolpidone to treat my insomnia ... Once I had a bad case of insomnia and I bought a 10 milligrams dose of Zolpidone from the pharmacy, which causes hallucination. Later on, I was hallucinating; I was seeing people whom my family could not see!!”* (Eighth interview).

##### Death

Some students were aware of this reality that sometimes the side effects of a drug could be so dangerous that could leads to death. The 15 codes were placed in this subcategory. Two of the participants talked about the deaths of people who used drugs without consulting doctor. One of the students said, *“One of my friends was an athlete, and used to inject insulin before sleep to increase his weight, he died one night after injecting insulin”* (Fifth Interview). Another student talked about the death of a woman who had used herbal medicine to treat infertility without a doctor’s advice: *“I knew a woman who could not have a baby and took herbal medicine to treat her infertility, but after the consumption of medicine she went into a deep coma and eventually died”* (Ninth Interview).

## Discussion

The present study aimed to explain the consequences of self-medication among nursing students. Self-medication had positive and negative consequences from the perspective of nursing students. One of the main and positive consequences was cost saving. Evidence suggests that one of the main reasons for self-medication in medical students is to save the cost [7, 14, 22]. The cost of treatment is high and cannot be paid by all people in the community. Despite all the strategies that have been put in place in Iran, including the health promotion plan, which has led to a significant reduction in the cost of treatment, unfortunately still part of the community practice self-medication to avoid paying the costs of treatment.

Time saving was another positive consequence of self-medication. Participants believed that, visiting a doctor takes a lot of time and with self-medication in simple diseases they could save time and money. Evidence suggests that, saving time is one of the important reasons for self-medication in nursing or medical students [4, 7, 14, 23–25]. Considering that, time is very important for students and due to the long process of doctor’s visit and long waiting time in Iran, it is recommended to use telephone or online systems to make appointment with doctor.

Another positive consequence of self-medication was the successful treatment of diseases. Most nursing students believed that, they could treat simple diseases. Evidence suggests that, medical students treat diseases such as fever, colds and pains without referring to a doctor and only by relying on previous experiences [14, 23, 24, 26–31]. We believe that, if self-medication is provided by qualified people with adequate medical information and knowledge about the side effects of drugs, it can be relatively safe in the treatment of simple diseases, but in complicated cases, referral to a physician is mandatory.

One of the negative consequences of self-medication was harming of the health system from the students’ point of view. Self-medication can impose a significant financial burden on limited resources of health system [32], which can be problematic for developing countries such as Iran, with relatively limited financial resources. According to the authors, by taking actions such as the provision and delivery of educational programs through mass media, significant steps can be taken to reduce the prevalence of self-medication and reduce the pressure on the health system.

Another perceived negative consequence of self-medication was drug resistance caused by taking some medications, especially antibiotics. Drug resistance is one of the most dangerous and relatively common side effects of antibiotic drugs, which can endanger the health of individuals [33–36]. Unfortunately, in Iran, one of the common reasons for self-medication is the easy access to many medications, even medicines requiring medical prescription such as antibiotics. We believe that, the country’s pharmacology authorities in the Ministry of Health should review the existing laws on drug sales in pharmacies.

The occurrence of physical complications and death was another negative consequence of self-medication according to the nursing students. In one study, 16.3% (No: 212) of self-medicating students developed side effects [13]. In another study, drug poisoning due to the simultaneous consumption of multiple drugs led to brain hemorrhage and ultimately death of an individual [12]. In this regard, arbitrary violation of the medications’ instruction of use can be dangerous. The risk of death in people who abuse drugs arbitrarily is significantly higher than those who use drug on a regular basis [37]. We believe self-medication can be used only for simple and common diseases such as colds and fever, and in other cases, self-medication can be dangerous and can lead to irreparable complications and even death.

The current study encountered a number of limitations. One of these limitations was the method of qualitative study that limits the generalizability of the results, and other is only nursing students were interviewed, therefore, it is suggested to conduct more studies on students in term of geographical background and educational fields in relation to the consequences of self-medication.

## Conclusion

The qualitative analysis resulted in two main categories and seven sub-categories. The main categories included perceived positive and negative consequences, and the sub-categories included time saving, cost savings, disease treatment, harming the health system, drug resistance, physical complications, and death. To avoid negative consequences, we need to pay attention to and plan the managers. Informing about the complications of self-medication, drug resistance, and vulnerability of the health system is important. However the codes related to the positive were more than negative, and regarding the students will change the society idea, it is suggested the students will be educated and warned about the adverse effects of self-medication in the schools and during training. Moreover, we recommend to carry out more studies about self-medication on other medical sciences students, as well as, planning a part of credit in educational curriculum of nursing related to self-medication and its consequences.

## Data Availability

Data available by contacting the corresponding author.

## References

[1] Limaye D, Limaye V, Krause G, Fortwengel G (2017). A systematic review of the literature to assess self-medication practices. Ann Med Health Sci Res.

[2] Osemene K, Lamikanra A (2012). A study of the prevalence of self-medication practice among university students in southwestern Nigeria. TJPR.

[3] Shehnaz SI, Agarwal AK, Khan N (2014). A systematic review of self-medication practices among adolescents. J Adolesc Health.

[4] Kumar N, Kanchan T, Unnikrishnan B, Rekha T, Mithra P, Kulkarni V (2013). Perceptions and practices of self-medication among medical students in coastal South India. PLoS One.

[5] Tabiei S, Farajzadeh Z, Eizadpanah A (2012). Self-medication with drug amongst university students of Birjand. ISO: Mod Care J.

[6] Ershadpour R, Marzouni HZ, Kalani N (2015). Review survey of the reasons of the prevalence of self-medication among the people of Iran. IJBMS.

[7] Ibrahim NK, Alamoudi BM, Baamer WO, Al-Raddadi RM (2015). Self-medication with analgesics among medical students and interns in king Abdulaziz University, Jeddah, Saudi Arabia. Pak J Med Sci.

[8] Jafari F, Khatony A, Rahmani E (2015). Prevalence of self-medication among the elderly in Kermanshah-Iran. Global J Health Sci.

[9] Hughes CM, McElnay JC, Fleming GF (2001). Benefits and risks of self medication. Drug Saf.

[10] Jain S (2011). Concept of self medication: a review. IJPBA.

[11] Zhao Y, Ma S (2016). Observations on the prevalence, characteristics, and effects of self-treatment. Front Public Health.

[12] Vacher R, Lagarce L, Ghamrawi S, Laugier-Castellan D, Vial T, Bagheri H (2020). Drug interactions related to self-medication: a French pharmacovigilance database study. Fundam Clin Pharmacol.

[13] Pan H, Cui B, Zhang D, Farrar J, Law F, Ba-Thein W (2012). Prior knowledge, older age, and higher allowance are risk factors for self-medication with antibiotics among university students in southern China. PLoS One.

[14] Dilie A, Gualu T, Haile D, Zuleta FA (2017). Knowledge, attitude and practice of self-medication among health science students at Debre Markos university, Northwest Ethiopia. J Public Health Epidemiol.

[15] Bengtsson M (2016). How to plan and perform a qualitative study using content analysis. Nurs lus Open.

[16] Abdi A, Faraji A, Dehghan F, Khatony A (2018). Prevalence of self-medication practice among health sciences students in Kermanshah, Iran. BMC Pharmacol Toxicol.

[17] Soroush A, Abdi A, Andayeshgar B, Vahdat A, Khatony A (2018). Exploring the perceived factors that affect self-medication among nursing students: a qualitative study. BMC Nurs.

[18] Kohan M, Borhani F, Abbaszadeh A, Sultan Ahmadi J, Khajehpoor M (2012). Experience of mothers with premature infants in neonatal. J Qual Res Health Sci.

[19] Erlingsson C, Brysiewicz P (2017). A hands-on guide to doing content analysis. Afr J Emerg Med.

[20] Adelmehrban M (2015). A review of qualitative content analysis and its application in research. 1, editor.

[21] Denzin NK, Lincoln YS (2011). The sage handbook of qualitative research: sage.

[22] Johnson M, Badyal DK (2018). Prevalence, knowledge, attitude and practice regarding self-medication among medical, dental and paramedical students in a tertiary care hospital. Int J Basic Clin Pharmacol.

[23] Mumtaz Y, Jahangeer S, Mujtaba T, Zafar S, Adnan S (2011). Self medication among university students of Karachi. Jlumhs..

[24] Banerjee I, Bhadury T (2012). Self-medication practice among undergraduate medical students in a tertiary care medical college, West Bengal. Postgrad Med J.

[25] Donkor ES, Tetteh-Quarcoo PB, Nartey P, Agyeman IO (2012). Self-medication practices with antibiotics among tertiary level students in Accra, Ghana: a cross-sectional study. Int J Environ Res Public Health.

[26] Badiger S, Kundapur R, Jain A, Kumar A, Pattanshetty S, Thakolkaran N (2012). Self-medication patterns among medical students in South India. Australas Med J.

[27] Gutema GB, Gadisa DA, Kidanemariam ZA, Berhe DF, Berhe AH, Hadera MG (2011). Self-medication practices among health sciences students: the case of Mekelle University. JAPS.

[28] Alshogran OY, Alzoubi KH, Farah S, Khabour OF (2018). Patterns of self-medication among medical and nonmedical university students in Jordan. Risk Manage Healthc Policy.

[29] Rathish D, Wijerathne B, Bandara S, Piumanthi S, Senevirathna C, Jayasumana C (2017). Pharmacology education and antibiotic self-medication among medical students: a cross-sectional study. BMC Res Notes.

[30] Kasulkar AA, Gupta M (2015). Self medication practices among medical students of a private institute. Indian J Pharm Sci.

[31] Sontakke S, Bajait C, Pimpalkhute S, Jaiswal K, Jaiswal S (2011). Comparative study of evaluation of self-medication practices in first and third year medical students. Int J Biol Med Res.

[32] Asseray N, Ballereau F, Trombert-Paviot B, Bouget J, Foucher N, Renaud B (2013). Frequency and severity of adverse drug reactions due to self-medication: a cross-sectional multicentre survey in emergency departments. Drug Saf.

[33] Sapkota AR, Coker ME, Goldstein RER, Atkinson NL, Sweet SJ, Sopeju PO (2010). Self-medication with antibiotics for the treatment of menstrual symptoms in Southwest Nigeria: a cross-sectional study. BMC Public Health.

[34] Nepal G, Bhatta S (2018). Self-medication with antibiotics in WHO southeast Asian region: a systematic review. Cureus.

[35] Rather IA, Kim BC, Bajpai VK, Park YH (2017). Self-medication and antibiotic resistance: crisis, current challenges, and prevention. Saudi J Biol Sci.

[36] Zaman SB, Hussain MA, Nye R, Mehta V, Mamun KT, Hossain N (2017). A review on antibiotic resistance: alarm bells are ringing. Cureus.

[37] Granger BB (2015). Self-reported medication adherence for heart failure is associated with lower risk of all-cause hospitalisation and death. Evid Based Nurs.

